# Lipid Metabolism–Signaling Crosstalk in Metabolic Disease and Aging: Mechanisms and Therapeutic Targets

**DOI:** 10.3390/nu17233699

**Published:** 2025-11-26

**Authors:** Paalki Sethi, Awdhesh Kumar Mishra, Shampa Ghosh, Krishna Kumar Singh, Samarth Sharma, Radoslav Stojchevski, Dimiter Avtanski, Jitendra Kumar Sinha

**Affiliations:** 1 GloNeuro, Sector 107, Vishwakarma Road, Noida 201301, India; 2 Department of Biotechnology, Yeungnam University, Gyeongsan 38541, Republic of Korea; awdhesh@ynu.ac.kr; 3 Symbiosis Centre for Information Technology (SCIT), Symbiosis International (Deemed University), Rajiv Gandhi InfoTech Park, Hinjawadi, Pune 411057, India; krishnakumar@scit.edu; 4 GL Bajaj Institute of Technology and Management, Greater Noida 201308, India; 5 Friedman Diabetes Institute, Lenox Hill Hospital, Northwell Health, New York, NY 10022, USA; rstojchevski@northwell.edu; 6 Feinstein Institutes for Medical Research, Manhasset, NY 11030, USA; 7 Donald and Barbara Zucker School of Medicine at Hofstra/Northwell, Hempstead, NY 11549, USA

**Keywords:** lipid metabolism, bioactive lipids, lipotoxicity, insulin resistance, aging, obesity, type 2 diabetes, MASLD (formerly NAFLD)

## Abstract

Lipid metabolism and lipid-derived signaling together ensure cellular and systemic homeostasis. Their dysregulation causes obesity, type 2 diabetes, cardiovascular disease, NAFLD/MASH, and neurodegeneration throughout life. This review integrates central pathways, such as ACC–FASN-mediated de novo lipogenesis, lipid-droplet lipolysis, and mitochondrial and peroxisomal β-oxidation, and their regulation by insulin–PI3K–Akt, glucagon–cAMP–PKA, SREBPs, PPARs, and AMPK. We emphasize the mechanisms by which bioactive lipids like diacylglycerols, ceramides, eicosanoids, and endocannabinoids serve as second messengers linking nutrient state to insulin signaling, inflammation, and stress response; pathologic accumulation of these species enhances insulin resistance and lipotoxicity. Aging disrupts these axes via diminished catecholamine-stimulated lipolysis, defective fatty-acid oxidation, mitochondrial failure, and adipose depot redistribution, facilitating ectopic fat and postprandial dyslipidemia. We suggest a pathway-to-phenotype paradigm that connects lipid species and tissue environment to clinical phenotypes, allowing for mechanism-to-intervention alignment. Therapeutic avenues range from lipid lowering for atherogenic risk to novel agents targeting ACLY, ACC, FASN, CPT1, and nuclear receptors, with precision lifestyle intervention in diet and exercise. Translation is still heterogeneous because of isoform-dependent effects, safety trade-offs, and inconsistent adherence. We prioritize harmonization of lipidomics with multi-omics for stratifying patients, enriching responders, and bridging gaps between mechanistic understanding and clinical outcome, with focus on age-sensitive prevention and treatment for lipid-mediated metabolic disease.

## 1. Introduction

Our lifestyle choices considerably impact our metabolic health, which can consequently result in a range of disorders [1]. Lipids, vital to metabolic processes, fulfil numerous essential functions within the human body. They act as structural components of cells, serve as energy reserves, participate in signaling pathways, function as biomarkers, contribute to energy metabolism, and serve as hormones. Disruption of these lipid-related processes may initiate a cascade of interconnected health issues, including diabetes, infections, as well as inflammatory and neurodegenerative diseases [2]. Lipids are organic compounds that don’t dissolve in water but do dissolve in organic solvents. These compounds are classic esters of fatty acids and sometimes include alcohol or phosphate functional groups. Lipids cover a range of molecules, such as triglycerides, phospholipids, and steroids. They serve as the primary energy reserves in animals, help regulate body temperature, form the essential components of cell membranes, and act as chemical messengers within the body [3]. The human body needs different types of beneficial fats to ensure its systems function properly. Maintaining a balance of lipid concentrations in the blood is crucial for overall health [4].

Lipid metabolism is essential for various vital functions, including energy storage, hormone regulation, neurotransmission, and the transport of fat-soluble nutrients. Lipids serve as a highly efficient energy source, providing 9 kcal per gram—much more than proteins and carbohydrates. The body can store up to 100,000 kcal of energy as lipids, enabling survival without food for 30–40 days with adequate water intake [5]. These biochemical lipids are stored mainly in cells, especially in adipose tissue, a type of connective tissue. They protect vital organs like the spleen, liver, heart, and kidneys by cushioning them from damage. Lipids in the blood are absorbed by liver cells, which distribute them to various parts of the body in appropriate amounts. The liver plays a central role in lipid metabolism, acting as a secondary storage reservoir for excess fats. When energy intake exceeds expenditure, the surplus is stored as triglycerides in adipose tissue and hepatocytes. The metabolic cycle also involves the citric acid cycle, the urea cycle, and the Krebs cycle [6]. Lipid metabolism plays a vital role in various cellular processes crucial for maintaining homeostasis, such as the synthesis of membranes and storing energy in the form of triglycerides (TG) [7]. Fatty acids (FAs) are vital lipids that make up the primary structural elements of membrane lipids, including glycerophospholipids (GPLs) and sphingolipids. Additionally, they serve as a key energy source through processes like mitochondria-mediated beta-oxidation and the tricarboxylic acid (TCA) cycle, also known as the citric acid cycle [8].

The classification of specific lipid metabolic disorders depends on the levels of various groups of lipoproteins. Many disorders are identifiable by structural defects, irrespective of the presence or absence of apolipoproteins and lipid transfer proteins [9]. Lipid metabolism issues or abnormalities can lead to a range of disorders and diseases. Excessive lipid storage in the body can result in conditions like xanthoma, Bassen-Kornzweig syndrome, chylomicronemia syndrome, familial lipoprotein lipase deficiency, Niemann-Pick disease (types A and B), methylmalonic acidemia, GM1 and GM2 gangliosidoses, and Gaucher disease. More serious consequences include cardiovascular disorders and diabetes, which often develop without noticeable symptoms. In today’s digital age, these have become major health concerns. The leading causes of acquired hyperlipidemia include diabetes mellitus, alcohol consumption, hypothyroidism, renal failure, and the prolonged use of diuretics and beta-blockers [10]. Variations in lipid levels can lead to lipid disorders, often characterized by elevated triglycerides or LDL (low-density lipoprotein), or both. The body relies on beneficial fatty acids like HDL (high-density lipoprotein) to remove bad cholesterol. Conversely, the buildup of harmful lipids such as LDL and triglycerides can damage arteries and increase cardiovascular risk. A recent study by Xiao et al. (2021) identified over 80 diseases associated with complex lipid metabolism defects [11], examining their role in health disorders, including nonlysosomal sphingolipids and acylceramides. Fredrickson’s classification divides lipid metabolism disorders into five types based on their pathways and health effects [12]. High amounts of circulating lipids have been associated with metabolic disorders and cancer. In metabolic disorders, an imbalance between the synthesis and expenditure of fatty acids (FAs) leads to the accumulation of lipid metabolites within cells. This build-up can cause dysfunctional cells and death in various tissues, involving the kidneys, brain, skeletal muscles, and heart [13].

As we age, body fat increases, accompanied by changes in lipid metabolism and metabolite levels. Surplus adiposity and amplified lipotoxicity contribute to many age-related diseases, such as cardiovascular disease, cancer, arthritis [14], type 2 diabetes, and Alzheimer’s disease [15]. Mechanistically, oxysterol stress can drive chondrocyte death via p53–Akt–mTOR signaling [16]. The complications of lipid metabolism create challenges to pinpoint its specific mechanisms and roles during aging. However, advances in genetic engineering techniques have shown that changes in lipid metabolism are linked to aging and related diseases. This review explores the latest progress in our understanding of lipid metabolism in aging, including conventional findings and new advances [17]. The objective of this review is to synthesize how core lipid pathways and lipid-derived signals intersect with metabolic disease and aging. We first outline lipid synthesis, storage, mobilization, and oxidation, then summarize enzymatic, hormonal, and transcriptional regulation. We next discuss how lipid-derived signaling integrates with these pathways, how their disruption contributes to cardiometabolic and neurodegenerative disorders, and how aging reshapes these interactions. Finally, we propose a pathway-to-phenotype framework that connects specific lipid signatures with therapeutic targets, spanning pharmacologic agents and lifestyle interventions across the lifespan.

In this review, fatty acid synthesis refers specifically to ACC–FASN–mediated chain elongation to palmitate, whereas lipogenesis denotes the broader process encompassing de novo fatty acid synthesis, triglyceride and phospholipid assembly, and cholesterol biosynthesis; lipolysis denotes hydrolysis of stored triglycerides to fatty acids and glycerol; β–oxidation refers to mitochondrial and peroxisomal fatty acid catabolism to acetyl–CoA for energy. Compared with existing reviews that separately address lipid metabolism, lipid signaling, or aging, we focus on their convergence into defined lipid lesions that can be mapped onto clinical phenotypes and therapeutic targets. This pathway-to-phenotype view emphasizes age-sensitive, tissue- and species-resolved interpretation of lipid data and is intended to guide mechanism-based intervention design.

## 2. Lipid Pathways: Synthesis, Storage, Mobilization, and Oxidation

The FAs play multiple critical roles in the body: they act as building blocks in cells, serve as central biochemical intermediates, influence membrane properties, modulate cellular signaling pathways, and provide a vital fuel source [18]. All lipids are water-insoluble; they can be broadly labelled as FAs, phospholipids, or neutral lipids (such as TGs and cholesteryl esters) [10]. FAs, the fundamental building blocks of all lipids, serve as precursors for the synthesis of more lipids, including glycerolipids, glycerophospholipids (GPLs), sphingolipids, sterols, and saccharolipids [19]. The biological roles of various lipid groups are determined by their lipid head groups. An imbalance between fatty acid absorption and oxidation can result in the buildup of long-chain fatty acids, which are subsequently incorporated into triglycerides, phospholipids, and other lipid species. Specific lipids, like ceramides, diacylglycerols (DAGs), and acyl-carnitines, are known to regulate intracellular signaling pathways and metabolism, but they are also considered toxic signaling lipid species [13].

### 2.1. Fatty Acid Synthesis

This process primarily occurs in the cytosol of hepatocytes and adipocytes, with elongation/desaturation in ER. Fatty acid production is an anabolic process that generates various lipid species. The enzyme fatty acid synthase (FASN) plays a key role in this process, altering dietary carbohydrates into long-chain saturated fatty acids, primarily generating the 16-carbon fatty acid palmitate. This conversion employs acetyl-CoA (Ac-CoA) as the primary building block. FASN is critical for supplying the required lipids that support the structure of cellular membranes and facilitate intracellular signaling. It begins with ATP citrate lyase (ACLY), an enzyme that converts citrate from the citric acid cycle (TCA) into Ac-CoA in the cytoplasm, which then acts as a precursor for fatty acid synthesis. Mitochondrial Ac–CoA cannot traverse the inner membrane. Because Ac–CoA cannot cross the mitochondrial inner membrane, citrate is exported via SLC25A1 and cleaved by ACLY to generate cytosolic Ac–CoA for fatty acid and cholesterol synthesis, with ACSS2 providing an acetate–to–Ac–CoA salvage route in cytosol. This complex metabolic pathway underlines the importance of lipid production in regulating cellular integrity and function [20]. In the process of fatty acid synthesis, FASN sequentially adds seven malonyl-CoA molecules to a single acetyl-CoA, producing palmitate, a 16-carbon saturated fatty acid. Once made, palmitate undergoes activation by acyl-CoA synthetase (ACS), which prepares it for further modification. The activated fatty acid can then be elongated by fatty acid elongase 5 (ELOVL5) and desaturated by enzymes like stearoyl-CoA desaturase (SCD) and fatty acid desaturase 2 (FADS2), providing a series of fatty acid molecules with distinct chain lengths and saturation degrees [21]. Synthesized fats are deposited in cells as TGs. Diglyceride acyltransferase (DGAT) performs a key role in the TG synthesis pathway by transforming DAG into triglycerides (TAGs) [22].

The enzymes participating in fatty acid synthesis are controlled at the transcriptional stage by sterol regulatory element-binding protein 1 (SREBP-1) transcription factors. Lately, a genome-wide CRISPR screen was conducted to systematically map genetic interactions (GIs) in human HAP1 cells (a near-haploid human cell line resulting from chronic myelogenous leukemia [CML]). This work aimed to explore how cells adapt to the loss of de novo FA synthesis [23].

### 2.2. Lipolysis

Adipose and liver lipid droplets are the primary sites and intracellular lipases act at the LD surface. Triglycerides serve as a proficient and inert form of FAs for storing and transporting, but they cannot cross cell membranes directly. Therefore, TGs must either be broken down into FAs and glycerol through hydrolysis or transported across cell membranes via specialized vesicles. This transport involves the secretion and uptake of TG-rich lipoproteins or the movement of TGs by extracellular vehicles (EVs). The breakdown of TGs, known as lipolysis, is catalyzed by lipases [24].

The function of neutral lipid metabolism in cellular signaling has gained significant attention, especially with the discovery that elevated TG levels in cells are closely linked to insulin resistance in muscle tissue and the liver [25]. However, the moderately inert nature of TGs suggests they do not directly interfere with insulin signaling. This idea is supported by the “athletes’ paradox”, where endurance athletes have higher TG accumulation in the lipid droplets (LDs) of their skeletal muscle cells yet remain highly insulin sensitive [26]. Similarly, mice lacking adipose triglyceride lipase (ATGL) accumulate large fractions of fat in several tissues, including skeletal muscle, heart muscle, liver, kidneys, and macrophages, but show increased insulin sensitivity. This increased sensitivity occurs despite ATGL deficiency, leading to an insulin-secretion defect in pancreatic islets [27].

At lipid droplets, ATGL activation via CGI–58 and PKA-phosphorylated HSL are coordinated by perilipins, while insulin restrains lipolysis through PDE3B-mediated dampening of PKA signaling. The G0S2 tonically inhibits ATGL, establishing a hormonal and protein–scaffold gate on FA release. Additionally, FAs actively contribute to cellular signaling pathways and the regulation of gene transcription. FAs or their products can bind to and activate nuclear receptors, a family of transcription factors that regulate genes responsible for energy homeostasis and inflammation. Among these, the peroxisome proliferator-activated receptors (PPARs) are the most explored. The PPAR family includes four members: PPARα, PPARγ-1, PPARγ-2, and PPARδ (also known as PPARβ). PPARα and PPARδ are abundantly expressed in oxidative tissues and control genes included in substrate delivery, substrate oxidation, and oxidative phosphorylation (OXPHOS). In contrast, PPARγ plays a key role in lipogenesis and lipid synthesis, with the highest expression levels found in white adipose tissue (WAT) [28].

According to recent advances, PPARs pose as complex therapeutic paradoxes that cause failures in clinical translational research. Studies show that PPARδ acts as a transcriptional repressor via direct RelA (p65) binding, leading to suppression of cytotoxic T lymphocyte functions, which may cause acceleration of cancer progression [29]. From a clinical perspective, mono-targeted PPAR advances have shown a critical gap in translation. For example, PPARα agonists, also known as fibrates, have actively failed in showing positive clinical results for metabolic liver disease treatment, despite their functional roles in lipid metabolism [30,31,32].

Elevated cellular levels of non-esterified fatty acids (FAs), especially palmitate, can lead to the production of lipotoxic lipids like ceramides, which disrupt effective insulin signaling. Furthermore, FAs can increase the formation of reactive oxygen species, which then activate redox-sensitive serine kinases. These kinases, in turn, impair insulin activity [33].

### 2.3. Lipogenesis

In humans, lipogenesis occurs in adipocytes and hepatocytes with organelle partitioning between cytosol and ER. The hepatic de novo lipogenesis can be quantitatively important in steatotic states even if adipose fat mass accrues largely from FA uptake. Most cells have the ability to transform carbohydrates into fatty acids, which are often stored as neutral lipids within lipid droplets. Increasing studies indicate that lipogenesis is important not only in metabolic tissues for maintaining overall energy balance but also in the immune and nervous systems for their growth, differentiation, and even in their pathological functions. Consequently, both excessive and insufficient lipogenesis are closely linked to disruptions in lipid homeostasis, potentially leading to conditions such as dyslipidemia, diabetes, fatty liver, autoimmune diseases, neurodegenerative disorders, and cancers. To ensure systemic energy balance, the enzymes involved in lipogenesis are tightly regulated through transcriptional and post-translational modifications [34]. Cytosolic acetyl–CoA for fatty acid and cholesterol synthesis derives from citrate exported by SLC25A1 and cleaved by ATP–citrate lyase (ACLY), positioning ACLY as a nodal enzyme between mitochondrial metabolism and lipogenesis. ACC carboxylates acetyl–CoA to malonyl–CoA, supplying FASN and constraining β-oxidation via CPT1 inhibition. On the other hand, SREBP–2 preferentially controls the cholesterol arm (e.g., HMG–CoA reductase) alongside SREBP–1 control of fatty acid genes. Citrate is exported via SLC25A1 and cleaved by ACLY to produce cytosolic acetyl–CoA for fatty acid and cholesterol synthesis, with acetate salvage via ACSS2 (Acyl–CoA synthetase short–chain family member 2) as an auxiliary source.

In rats, lipogenesis primarily takes place in the liver and WAT, while in humans, it plays a minor role in overall fat balance. Lipid accumulation in adipose tissue largely depends on the uptake of circulating FAs. These FAs are released through the enzymatic breakdown of TG in chylomicrons by lipoprotein lipase. Once FAs enter the adipocyte, they must be re-esterified for storage in the form of TG. Several enzymes involved in adipose tissue lipogenesis, such as fatty acid synthase (FAS), acetyl-CoA carboxylase (ACC), and malic enzyme (ME), are induced by insulin. Newly synthesized FAs are then utilized as substrates for TG synthesis. The insulin-mediated stimulation of lipogenesis in response to nutritional status results from increased enzyme activities implicated in FA biosynthesis and elevated gene expression of these enzymes [35]. In skeletal muscle, de novo lipogenesis and re-esterification can operate alongside fatty acid oxidation. And this is known to generate a ‘futile’ lipid cycle that dissipates energy and buffers lipid intermediates, particularly under chronic stress or denervation-associated catabolism [36,37,38]. This cycling is heightened when substrate influx and oxidative demand are simultaneously elevated. This is observed in neuromuscular disease models. Emerging data indicate that neuronal metabolites such as N-acetylaspartate (NAA) can contribute acetate equivalents to peripheral acetyl-CoA pools via aspartoacylase and acetyl-CoA synthetase, potentially augmenting myocellular lipogenesis and re-esterification under high turnover states, thereby intensifying futile lipid cycling in disease contexts [39,40].

### 2.4. β-Oxidation

Mitochondria are the primary site in oxidative tissues and peroxisomes for the very-long-chain substrates in our body. Long-chain fatty acids are initially activated by acyl-CoA synthetases and transported into mitochondria by the carnitine shuttle (CPT1/CACT/CPT2). On the other hand, very-long-chain species undergo initial peroxisomal chain–shortening before mitochondrial oxidation, with tissue differences in flux partitioning across liver, heart, and skeletal muscle. CPT1 isoforms integrate malonyl–CoA inhibition, coupling β-oxidation to lipogenesis state, while glucagon–PKA signaling lowers malonyl–CoA and promotes oxidation via CPT1 induction, establishing a hormonal gate at the mitochondrion. Further dehydrogenation, hydration, and thiolysis produce acetyl-CoA for the tricarboxylic acid cycle [41]. Although skeletal muscle and cardiac oxidation play a key role in regulating energy homeostasis under aerobic conditions, adipose-specific loss does not affect full-body insulin sensitivity. Moreover, tissue-specific knockout models of carnitine palmitoyl transferase 2 indicate that organs act differently for metabolic functions [42].

Pathway-to-tissue mapping helps in clarifying the phenotypes. Like, adipose lipolysis controls FA delivery and the hepatic de novo lipogenesis determines VLDL output. Additionally, cardiac/skeletal mitochondrial β-oxidation is known to govern the fasting endurance, while peroxisomal oxidation safeguards against very-long-chain lipid toxicity.

## 3. Regulation of Lipid Metabolism: Enzymatic and Hormonal Control

### 3.1. Enzymatic and Hormonal Control

Insulin is a major regulator of lipid metabolism and energy homeostasis. This role is especially significant in individuals with insulin resistance, where the liver tends to accumulate fat due to enhanced de novo lipogenesis. Studies have indicated the importance of the phosphoinositide 3-kinase (PI3K)-Akt pathway as a crucial signaling mechanism through which insulin exerts its metabolic effects [43]. When insulin binds to its receptor, it initiates the recruitment of PI3K, which then phosphorylates insulin receptor substrates (IRS). This phosphorylation makes 3′-phosphoinositides, causing the formation of phosphatidylinositol (3,4,5)-trisphosphate (PIP3). PIP3 facilitates the recruitment of pyruvate dehydrogenase kinase 1 (PDK1) and Akt (protein kinase B). PDK1 phosphorylates Akt, a process also influenced by mammalian target of rapamycin complex 2 (mTORC2) at a different site on Akt. Once activated, Akt triggers several downstream pathways, including mTORC1, glycogen synthase kinase, and FoxO transcription factors, all of which play important roles in regulating glucose and lipid metabolism [44].

### 3.2. Transcriptional and Epigenetic Control

Hormonal cues are known to converge on the transcriptional programs. For example, insulin–Akt promotes SREBP1c processing, whereas fasting hormones and AMPK suppress lipogenesis and favor oxidation. This helps in integrating rapid signaling with gene–level reprogramming. Sterol regulatory element-binding proteins (SREBPs) are a class of transcription factors essential for the synthesis of fatty acids, triglycerides (TGs), cholesterol, and its esters. Insulin activation of Akt enhances both the synthesis and processing of SREBP1c, the predominant SREBP subtype in liver cells. During the processing of the SREBP1c precursor, insulin increases its affinity for vesicles and the Golgi apparatus and enhances its proteolytic cleavage. This cleavage, induced by SREBP cleavage-activating protein (SCAP), is dependent on the PI3K-Akt pathway [45].

Glucagon predominantly acts on hepatocytes, which express the highest levels of glucagon receptors. Upon binding to these receptors, glucagon triggers the accumulation of cyclic AMP (cAMP). CAMP-response element-binding protein (CREB) is further activated by this, increasing carnitine acyltransferase (CPT-1) transcription. The primary function of CPT-1 is to convert fatty acids into acylcarnitine, which promotes fatty acid breakdown and facilitates β-oxidation [46]. Additionally, glucagon binding induces PKA-dependent phosphorylation, which inactivates acetyl-CoA carboxylase, a significant enzyme in malonyl-CoA synthesis. Meanwhile, malonyl-CoA inhibits β-oxidation by suppressing CPT-1 activity. Reducing malonyl-CoA levels allows additional free fatty acids to enter mitochondria for β-oxidation instead of being re-esterified and then released into the bloodstream as VLDL from hepatocytes. Generally, glucagon is sufficient to activate fatty acid oxidation gene expression, while the insulin-PI3K-Akt pathway inhibits Foxa2 through phosphorylation at the Thr156 site and a nuclear exclusion mechanism [47].

Hormone levels fluctuate over time, such as the significant increase in growth hormone (GH) during adolescence; similarly, estrogen and progesterone in women at reproductive age. The maintenance of lipid homeostasis also varies depending on whether the body is in a fed or fasted state. Providing the complexity of the regulatory network in vivo, assessing the influence of individual hormones on animal models is extremely challenging [48]. Nevertheless, understanding the hormonal regulation of lipid metabolism is crucial. This knowledge helps us comprehend metabolic phenotypes characterized by hormone excess or deficiency and paves the way for developing new targeted therapies that can modulate hormone levels effectively [43].

## 4. Interconnections Between Lipid Metabolism and Signaling Pathways

Lipid metabolism and signaling pathways are strongly interconnected by reciprocal feedback mechanisms. Metabolites, primarily DAG and phosphatidylinositol, act as key secondary messengers in signaling cascades. For instance, phosphatidylinositol-4,5-bisphosphate (PIP2) is hydrolyzed by phospholipase C, producing DAG and inositol triphosphate (IP3). These molecules respectively activate protein kinase C and calcium signaling, which are essential pathways affecting metabolism, growth, and cell survival [49]. Likewise, SREBPs and liver X receptors play a role in lipid biosynthesis by acting as lipid-sensing transcription factors to respond to intracellular lipid levels [50]. PPAR agonists show strong preclinical efficacy but mixed clinical translation in NASH/MASH; while the pan–PPAR agonist lanifibranor improved histology and fibrosis in recent trials, historical α/δ/γ-targeted agents produced variable results and safety trade-offs, highlighting isoform- and tissue-selective actions and off-target liabilities that complicate bedside translation [32,51].

Single-cell lipidomics reveals lineage- and state–specific lipid programs that instruct fate decisions and stress responses, while spatial lipidomics links metabolite gradients to microanatomy; integrating these layers with single-cell RNA-seq and proteomics should identify lipid checkpoints actionable by pathway–selective drugs and enable biomarker panels for responder enrichment. Priority needs include standardized acquisition/annotation and robust cross–platform harmonization to ensure reproducibility and regulatory acceptability in clinical pipelines [52,53]. Recent single-cell and spatial lipidomics studies in atherosclerotic plaque, fatty liver disease, and solid tumors show that lipid species and signaling nodes are highly cell-type and niche-specific, refining links between ceramide or oxysterol enrichment and inflammatory or fibrotic phenotypes. Joint analysis with single-cell transcriptomes and proteomes is beginning to reveal actionable checkpoints and composite biomarkers that can stratify patients for therapies targeting de novo lipogenesis, sphingolipid metabolism, or nuclear receptors. These approaches also explain why pathway-level interventions may yield tissue-divergent effects and underscore the need for responder enrichment based on lipidomic context.

Metabolic signaling molecules derived from lipids also serve as stress-responsive cellular modulators. Lipid-derived factors, such as eicosanoids, function as signaling molecules that modulate inflammation, apoptosis, and oxidative stress [54]. Furthermore, peroxisome proliferator-activated receptors (PPARs) are stimulated by several fatty acids and regulate genes required in β-oxidation, inflammation, and lipogenesis, governing metabolic adaptations during stress [55].

The pathogenesis occurs when there is a disruption in the equilibrium between lipid signaling and metabolism. For instance, excessive lipid accumulation increases ceramide and DAG levels in obesity and type-2 diabetes, affecting insulin receptor signaling and causing insulin resistance [56]. Similarly, metabolic syndrome shows altered endocannabinoid signaling, which affects appetite, glucose, and lipid homeostasis [57]. In the nervous system, the dysregulation of lipid signaling pathways hinders neuronal function and survival, playing a role in neurodegenerative diseases like Alzheimer’s and Parkinson’s [58,59]. The harmful effects of disrupted lipid signaling highlight the importance of a balanced metabolic-signaling axis for maintaining health [60].

## 5. Dysregulation of Lipid Metabolism in Metabolic Disorders

Fluctuations in lipid levels can lead to various health issues known as lipid disorders. These disorders often manifest as elevated triglyceride and LDL cholesterol levels. The body relies on high-density lipoprotein (HDL) to carry bad cholesterol out of the bloodstream. However, the accumulation of harmful lipids, such as LDL cholesterol and triglycerides, can impair arteries and pose significant risks to cardiovascular health [2]. According to Xiao et al. (2021), they identified over 80 diseases related to defects in lipid metabolism [11]. Recent work links disease progression to lipid-metabolic reprogramming in MASLD and cancer [61]. Hyperlipidemia refers to a group of critical lipid disorders characterized by abnormally high levels of unwanted lipids in the blood [62]. These disorders are classified based on the concentrations of different classes of lipoproteins. Additionally, several lipid metabolic disorders are now identifiable by structural defects, which can occur with or without the presence of apolipoproteins and lipid transfer proteins, as mentioned in Table 1. Recent human cohort analyses link circulating ceramide panels to insulin resistance and adiposity indices, providing primary evidence for lipid-species–level risk stratification in metabolic disease. In MASH, pan–PPAR agonism with lanifibranor improved histological endpoints in phase 2 studies, supporting translational potential under careful safety surveillance [32,63].

Beyond taxonomy, these disorders demarcate therapeutic windows. For example, ceramide-centric phenotypes may benefit from sphingolipid-axis modulation, whereas hypertriglyceridemic states implicate DAG–PKC signaling and lipoprotein remodeling. This further reinforces the need for lipid-species–resolved diagnostics to match mechanism with therapy [70,71,72].

Mechanistically, ceramide accrual impairs Akt signaling and promotes insulin resistance, while DAG-driven PKC activation diminishes proximal insulin receptor signaling. And this together fuels hyperinsulinemia as well as steatosis. Aligning these lesions to therapeutic axes (e.g., sphingolipid modulation and PPAR targeting) can improve matchmaking of interventions to dominant biology. In MASH, pan–PPAR agonism shows histological gains with safety considerations, reinforcing mechanism-guided selection and monitoring. These pathway-level disturbances define phenotypes that should guide the selection and intensity of lipid-targeted pharmacologic and lifestyle interventions, developed further in Section 6.

## 6. Aging and Lipid Metabolism/Signaling

Aging is a complex physiological process described by the accumulation of aggregation over time, leading to a decline in physiological integrity [73]. These changes often increase susceptibility to specific age-related diseases. Although significant advances in studies have expanded our knowledge of aging and associated diseases, the fundamental causes of aging remain elusive due to its intricate and multifaceted nature [17]. Lipid metabolism undergoes significant changes as we age, affecting lipid content in tissues and the inter-tissue transport of lipids. Aging leads to higher concentrations of plasma triglycerides and elevated plasma lipoproteins, while the clearance rate of plasma triglycerides declines due to reduced activity of lipoprotein lipase [74]. Recent findings have shed light on the mechanisms behind these changes, showing that aging reduces lipolysis, partly due to reduced accessibility of catecholamines in adipose tissue. Additionally, the action of hormone-sensitive lipase, which is typically stimulated by β-adrenergic receptors on adipocytes, diminishes in aged adipose tissue. These alterations contribute to increased adiposity in both adipose tissue and plasma. These systemic lipid-handling changes are compounded by organ-specific alterations that further characterize the aging phenotype [75].

As with other tissues, aging brings significant changes to adipose tissue. Remarkably, obesity and aging share comparable mechanisms and effects on this tissue [76]. Since adipose tissue impairment impacts the entire body, it has become a key focus in aging intervention studies. Aging leads to notable shifts in adipose tissue biology, including alterations in abundance, allotment, and cellular constitution [77]. These alterations affect the tissue’s endocrine functions, influencing insulin resistance and metabolic dysfunction. Despite sex differences in fat deposition, both men and women typically experience increases in body mass and fat percentage as they age. A major variation in aging adipose tissue is fat redistribution, with fat shifting from subcutaneous to visceral depots. This shift is crucial because visceral fat accumulation is linked to a higher chance of metabolic syndrome, whereas subcutaneous fat accumulation is linked with a lower disease risk [78]. Given its systemic significance, adipose tissue has been implicated to play a primary role in longevity [79]. Various studies have shown that lifespan-extending interventions significantly impact adipose tissue biology. These anti-aging interventions include dietary restriction models (such as caloric restriction), gene mutation models targeting the GH/IGF-1 axis, and pharmacologic interventions like metformin and resveratrol [80].

Aging skeletal muscle displays a metabolic shift toward oxidative phenotypes with increased dependence on lipid catabolism and mitochondrial respiration. Yet this is accompanied by impaired mitochondrial quality control and heightened ROS [81], which together degrade contractile performance and endurance. Redox stress also impairs satellite cell regenerative capacity and alters redox–sensitive signaling cascades implicated in sarcopenia, linking mitochondrial dysfunction to progressive weakness [82]. Oxysterol-induced oxidative stress and regulated cell death provide another mechanistic link between disturbed lipid homeostasis and age-related tissue injury [16]. Together, these alterations across adipose and muscle tissues highlight the systemic remodeling of lipid metabolism during aging, predisposing older adults to metabolic and functional decline.

### 6.1. Alterations in Lipid Signaling Pathways During Aging

Beyond their classical role in energy homeostasis, lipid metabolites have various other functions, such as participating in signaling pathways and serving as structural constituents of cell membranes. These roles often include specific types of fatty acids or other lipid metabolites. Notably, several PUFAs are crucial in cellular signaling pathways [83]. Recent studies have started to discover the molecular connections between lipid metabolism and longevity by characterizing pro-longevity signaling pathways. This review focuses on three extremely conserved mechanisms that regulate longevity: insulin/IGF-1 signaling (IIS), mechanistic target of rapamycin (mTOR) signaling, and germline endocrine signaling. These pathways play crucial roles in the complex relationship between lipid metabolism and lifespan extension [10].

A persistent controversy concerns whether ceramides or DAGs are the dominant lipid drivers of insulin resistance across tissues and phenotypes. This is because ceramides inhibit Akt via PP2A activation and PKCζ-dependent sequestration, whereas DAGs can activate novel and conventional PKCs to impair insulin receptor signaling, with effect sizes that vary by lipid species, muscle fiber type, and adiposity state. Recent human and rodent work indicates that very-long-chain ceramide species and specific DAG–PKC axes associate with insulin resistance in a context-dependent manner, cautioning against single-lipid explanations and underscoring the need for species-, tissue-, and lipid-species-resolved analyses [70,71,72].

IIS: The daf-2 gene encodes the insulin/IGF-1 receptor, while age-1 encodes the downstream phosphoinositide 3-kinase. The pro-longevity outcomes of these mutations are entirely dependent on daf-16, a FOXO transcription factor. Subsequent research has confirmed that this longevity-regulating pathway is highly conserved across fruit flies, mice, and humans [84]. Interestingly, some long-lived daf-2 mutant alleles exhibit increased fat storage, though this is not true for all such mutants. Further analyses have shown that different site-specific mutations can differentially impact both longevity and fat storage. Therefore, the extended lifespan observed with reduced IIS is not merely due to an increase in overall fat storage but likely results from specific alterations in lipid metabolism [85]. Despite the widespread expression of the DAF-16/FOXO transcription factor, its role in regulating longevity is tied explicitly to its activity in fat storage tissues. In C. elegans, this effect is mediated by the DAF-16 target mdt-15, which encodes a transcriptional mediator subunit involved in this regulation [86].

In investigating the lipid intermediates that contribute to insulin resistance, ceramides and DAGs have been identified as essential bioactive lipids, exhibiting both divergent and occasionally opposing effects on insulin signalling pathways. Ceramides hinder insulin functionality by activating protein phosphatase 2A and facilitating PKCζ–mediated inhibitory phosphorylation of Akt, consequently impairing glucose uptake and metabolic regulation [87].

mTOR: Another key mechanism regulating longevity involves mTORC1, a complex made up of three core parts: Raptor, mLST8, and mTOR kinase. mTORC1 acts as a crucial operator of growth and metabolism, manipulated by growth factors, energy levels, and nutrient accessibility within the cell [88]. Growth factors like insulin and IGF-1 activate mTORC1 through their associated kinases. In contrast, low energy levels inhibit mTORC1 activation via the AMP/ATP sensor, AMPK. Nutrients such as amino acids, cholesterol, and glucose activate mTORC1 by promoting its localization to lysosomes and activating it through Rheb. Once activated, mTORC1 regulates various downstream effectors, including ribosomal S6 kinase (S6K), TFEB, SREBP, and Nrf2 transcription factors. Reduced mTORC1 signaling has been shown to extend lifespan in C. elegans, particularly when let-363/mTOR is knocked down. Similar lifespan-extending effects are observed when components of the mTORC1 signaling pathway are reduced, such as Rag GTPases (raga-1 and ragc-1), rheb-1/Rheb, daf-15/Raptor, and rsks-1/S6K [89]. mTORC1 signaling is pivotal in regulating various aspects of lipid metabolism, including lipogenesis, lipolysis, lipophagy, and fatty acid β-oxidation. As a central regulator of anabolic processes, mTORC1 activation drives lipid biogenesis by activating the SREBP transcription factor, which in turn upregulates genes involved in lipogenesis [90]. On the other hand, various cellular processes are influenced by mTOR signalling such as cell survival, autophagy, and protein synthesis. The upstream regulators, including insulin/IGF1, AMPK, PI3K, and amino acids, hyperactivate mTORC1, which suppresses autophagy, resulting in the accumulation of neurotoxic components, such as amyloid-β and neurofibrillary tangles [91].

**Figure 1:**
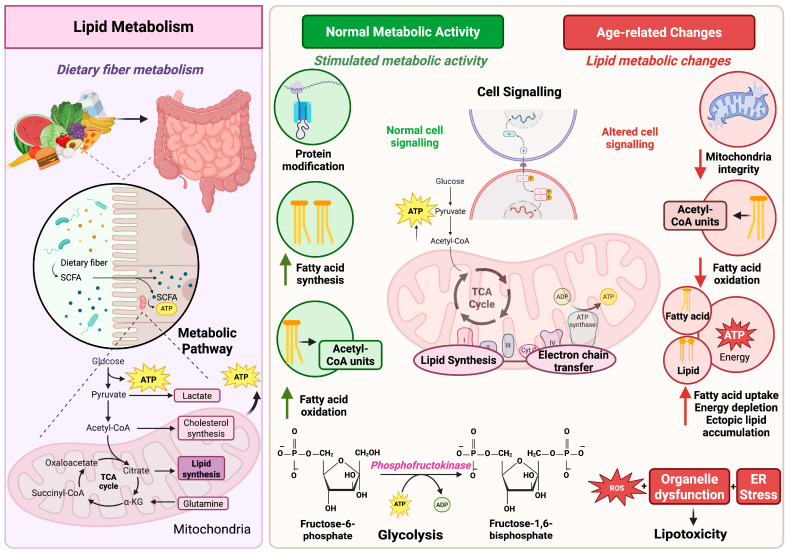
Overview of lipid metabolism, energy regulation, and age-related metabolic changes. Dietary fiber is metabolized by gut microbiota into short-chain fatty acids (SCFAs), contributing to energy production and lipid metabolism. Lipid pathways include glycolysis, the TCA cycle, and mitochondrial synthesis, supporting cell signaling, protein modification, and fatty acid oxidation. In healthy states, cytosolic acetyl–CoA for lipid and cholesterol synthesis derives from citrate export and ACLY cleavage, while mitochondrial acetyl–CoA feeds the TCA cycle. Aging disrupts lipid metabolism, leading to reduced mitochondrial integrity, impaired acetyl-CoA production, and decreased fatty acid oxidation. These changes result in ectopic lipid accumulation, ATP depletion, and increased oxidative and ER stress, causing lipotoxicity. Created in BioRender. Singh, D. (2025) https://BioRender.com/0qices2.

Lipids serve as fundamental signaling units that can regulate nuclear transcription and cellular interaction. Recent studies have highlighted their active role in longevity, the importance of lipid-binding proteins in transporting hydrophobic lipids through aqueous environments, and the involvement of receptors in identifying and transducing lipid signals (Figure 1).

FAs and their derivatives influence longevity through several signaling pathways. For instance, increased production of monounsaturated fatty acids (MUFAs) has been linked to longer lifespan in C. elegans through insulin/IGF-1 signaling reduction and germline loss, with oleic acid possibly enhancing longevity by activating the SKN-1/Nrf2 transcription factor, which is known for controlling oxidative stress responses [92]. However, the exact mechanism remains unknown. Omega-3 fatty acids like α-linolenic acid (ALA) extend lifespan in C. elegans via SKN-1, but indirectly through its oxidation products rather than directly. Moreover, fatty acyl ethanolamides (NAEs), which involve compounds like anandamide and oleoylethanolamide, have different effects on longevity. While some NAEs, like oleoylethanolamide, promote longevity, others, such as eicosapentaenoylethanolamide, might reduce it. The mechanisms by which these lipids affect lifespan, including their interactions with specific receptors and transport proteins, are still not well understood [93].

### 6.2. Age-Related Metabolic Disorders and Association with Lipid Dysfunction

Aging significantly raises the risk of chronic diseases, with over half of older adults affected by at least one such condition. Cardiovascular disease (CVD) impacts up to 70% of the elderly, while diabetes impacts around 20%. The financial and health burdens of these diseases are substantial and are expected to increase. Preventing these conditions early on is crucial. Since these diseases often involve dyslipidemia and insulin resistance due to lipid metabolism issues, targeting lipid metabolism might be a promising approach to prevent or manage these chronic health problems [94]. Aging affects all aspects of gastrointestinal function, including movement, enzyme activity, and hormone release, which further impacts digestion and nutrient absorption, leading to decreased nutrient uptake [95,96]. Postprandial lipemia (PPL), the rise in blood lipids after meals, tends to increase with age despite reduced intestinal absorption. This suggests that, even with decreased uptake, lipid levels in the blood remain elevated after eating, indicating a disruption in lipid circulation. Proper regulation of lipid metabolism post-absorption is essential for maintaining lipid balance. Age-related changes in lipid synthesis and breakdown result in abnormal lipid usage in tissues, leading to elevated blood lipids and increasing the risk of chronic impairments such as CVD, type 2 diabetes (T2D), non-alcoholic fatty liver disease (NAFLD), and obesity. Lipid-driven oxidative stress and chronic low-grade inflammation are also implicated in age-related tissue degeneration, including osteoarthritis [14]. This underscores the significance of regulating lipid metabolism to support overall health in the elderly [97].

In amyotrophic lateral sclerosis, systemic hypermetabolism can reflect increased lipid catabolism and mitochondrial uncoupling in thermogenic adipose tissues, with experimental models showing early and persistent activation of brown adipose tissue and browning in white depots, increased UCP1 expression and augmented β-oxidation that divert substrates into heat [98]. This thermogenic drain worsens negative energy balance and muscle wasting, suggesting that tailored nutritional support and strategies that modulate adipose thermogenesis may complement neuroprotective approaches.

Excessive dietary fat intake can lead to lipid buildup in the body, increasing the risk of chronic lipid-related dysregulations. Age-related changes in lipid absorption are often due to declines in digestive tract function, such as atrophy of tissues, decreased pancreatic secretions, lower lipase production, and reduced bile acid levels. Research shows that while lipid absorption may diminish with age, cholesterol absorption tends to do otherwise. As a result, focusing on lipid digestion and absorption could be a promising strategy for dietary interventions aimed at preventing dyslipidemia and managing lipid-related diseases in older adults [99]. As we age, one major alteration in pancreatic function is the reduction in pancreatic lipase action. In healthy individuals, pancreatic lipase is crucial for digesting about 75% of dietary triglycerides. However, its efficiency diminishes with age. Studies have shown that both the level and secretion volume of lipase gradually decrease after the age of 30 years. For instance, researchers analyzed duodenal fluid from elderly patients without pancreatic diseases after continuous intravenous infusions of irritants. They found a significant decrease in lipase concentration, about 15% lower in the older group compared to younger individuals. Moreover, lipase output levels dropped significantly by 45%. Similar findings were detected in aging mice, where pancreatic lipase function significantly decreased [100].

#### 6.2.1. Cardiovascular Disease (CVD)

CVD is the leading cause of death among the elderly and is also often regarded as the disease of aging. Atherosclerosis, which is strongly linked to lipid metabolism disorders, plays an essential role in CVD. As people age, disruptions in lipid metabolism lead to increased concentrations of cholesterol, TGs, and LDLs in the blood [101]. This results in lipid deposits forming under the inner lining of blood vessels, creating atherosclerotic plaques. Research has shown a positive correlation among levels of LDL cholesterol, total cholesterol (TC), and TGs with the prevalence of coronary heart disease. Notably, the TC to HDL-C ratio is the strongest indicator of coronary heart disease in older adults. Therefore, it is crucial to continuously monitor these clinical markers as we age [102].

As we age, there’s a higher tendency for deposition of lipids in the heart and blood vessels. For example, mice aged 25 months exhibit significantly higher triacylglycerol accumulation in their heart tissues compared to the 4-month-old mice [103]. Older mice also show increased lipid deposits in their aortic arches. Research has identified that the cluster of differentiation 73 (CD73), an exonuclease that converts adenosine monophosphate (AMP) to adenosine, can promote atherosclerosis in aged mice by inhibiting lipid catabolism. CD73 appears to accelerate excess vascular lipid accumulation, contributing to atherosclerosis [104].

#### 6.2.2. Type 2 Diabetes (T2D)

T2D is a common metabolic disorder among elderly people, characterized by impaired lipid metabolism that causes abnormal lipoprotein levels and contributes to diabetes progression. Research on older Chinese adults has shown an inverse relationship between HDL-C levels and diabetes risk, while the triglyceride-to-HDL-C ratio is positively correlated with T2D risk. Other lipid-linked markers also serve as predictors for T2D. Individuals with T2D typically exhibit a higher visceral adiposity index, which can help predict the risk of developing the disease. The triglyceride-to-glucose fasting index is another useful measure for diagnosing prediabetes in the elderly [105,106].

Lipid metabolism diseases mainly impact glucose metabolism through insulin resistance, which is a crucial factor in diabetes development. Abnormal lipid metabolism can lead to ectopic fat storage, promoting insulin resistance. Along with intolerance for glucose, insulin resistance triggers T2D as people age [107]. Studies have found that postprandial triglyceride levels are higher in individuals with T2D compared to those without, due to insulin resistance in the intestinal epithelial cells. To address this, researchers suggest focusing on chylomicron metabolism to enhance postprandial blood lipid and glucose levels in T2D patients [108].

#### 6.2.3. Obesity

The rising obesity rates among the elderly are a significant concern, as obesity accelerates aging and increases the risk of age-associated diseases, mainly sarcopenia, T2D, CVD, and other lipid-related conditions [109]. Research shows that obesity in older adults is linked to lipid metabolism disorders. As people age, the activity of the sympathoadrenal system decreases, which may slow lipid turnover rates and contribute to obesity [110]. Aging also affects the supply and function of adipose tissue. Older adults often accumulate more visceral fat and lose subcutaneous fat, with a rise in white adipose tissue size and a reduction in brown adipose tissue function [111]. In older rats, adipocytes become larger, show reduced de novo lipogenesis (DNL) and lipolysis, and exhibit increased esterification. Additionally, the mass and heat-producing capacity of brown adipose tissue diminish with age, leading to fat accumulation and higher obesity rates in elderly people [112].

Adipose-derived stromal/stem cells (ASCs) are crucial for maintaining energy balance, fat storage, and adipocyte homeostasis. However, the capacity of ASCs to proliferate and differentiate declines with age. Findings have suggested that transplanting ASCs from young mice into older mice improved the stem cell plasticity, liver function, and lipid metabolism of older mice. This indicates that ASCs could have potential as anti-aging and anti-obesity treatments [113].

#### 6.2.4. Non-Alcoholic Fatty Liver Disease (NAFLD)

NAFLD is a substantial age-related issue characterized by the accumulation of excessive lipid droplets in liver cells, known as simple steatosis. This condition can progress to non-alcoholic steatohepatitis (NASH) and associated fibrosis [114]. In Asia, research indicates that NAFLD affects around 40% of the elderly population and can mature into liver fibrosis with age. Similarly, it has been observed in aging mice, where TG and cholesterol levels increase in the liver as they age [115].

Research suggests that aging promotes NAFLD through elevated lipid synthesis and reduced lipid breakdown in the liver. Dietary fat intake also highly impacts liver fat in older mice [115]. Under a high-fat diet (HFD), aged mice show higher levels of neutral fat in their livers compared to younger mice. The levels of liver-protective polyunsaturated fatty acids (FAs) decrease, while the levels of lipotoxic saturated and monounsaturated FAs increase [116]. Finding efficient therapeutic targets for age-associated NAFLD is crucial. One potential target is CDGSH iron-sulfur domain-containing protein 2 (Cisd2), which decreases by about 50% in the livers of aged mice, leading to increased hepatic fat synthesis and accumulation. Enhancing Cisd2 levels could help slow liver aging [117]. Another potential target is the zinc finger gene 1 (JAZF1). In aged mice, those with JAZF1 overexpression show reduced hepatic lipid deposition, likely due to decreased expression of genes involved in fat storage, such as SREBP-1c, SCD-1, and fatty acid synthase (FAS) [118]. Targeting JAZF1 may help prevent excessive lipid production in the liver.

Species and tissue divergences in lipid handling (e.g., peroxisomal vs. mitochondrial β-oxidation balance, adipose lipolysis dynamics and hepatocyte lipogenesis set–points) limit direct extrapolation from animal models. Therefore, emergent single–cell and spatial lipidomics in human samples can contextualize biomarkers to cell types and microenvironments, which would further improve the clinical relevance [52,53].

These age-associated alterations in lipid handling and tissue crosstalk define distinct vulnerability profiles that should inform selection and intensity of lipid-targeted pharmacologic and lifestyle interventions, as outlined in the following section.

## 7. Therapeutic Approaches Targeting Lipid Metabolism and Signaling

**Figure 2:**
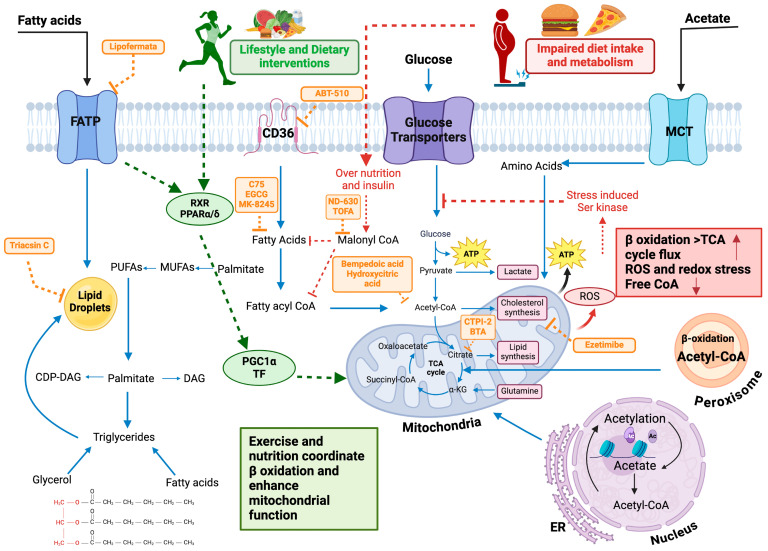
Integrated map of lipid pathways and therapeutic entry points. The diagram summarizes de novo lipogenesis (DNL), triglyceride synthesis/storage, lipolysis, fatty-acid oxidation (FAO), and lipid-derived signaling. Pharmacologic targets are indicated next to their primary pathways (e.g., ACLY/ACC/FASN along DNL; DGAT and MTTP in triglyceride synthesis/VLDL export; PCSK9 in LDL receptor cycling), while lifestyle levers (dietary pattern, weight loss, aerobic and resistance training) act across multiple nodes by reducing DNL substrate flux, improving insulin action, and enhancing FAO. Created in BioRender. Singh, D. (2025) https://BioRender.com/o1ih5el.

Given heterogeneous responses and class-specific safety considerations, translational implementation depends on aligning mechanism with patient phenotype, regulatory endpoints, and long–term adherence feasibility. Currently, our understanding of lipid metabolism mechanisms is still in its early stages. Lately, several anti-tumor drugs targeting lipid metabolism have been introduced, with some demonstrating substantial anti-tumor properties. The key challenge is to enhance the specificity of the inhibitors to target cancer cells without disrupting normal cellular metabolism. For instance, CPT1, a crucial enzyme in the FAO pathway, has several roles and tissue distributions among its three isoforms. This review highlights the research progress on several important targets and their inhibitors within the lipid metabolism process, given in Table 2, emphasizing the need for specific and safe therapeutic strategies (Figure 2) [119]. Despite promising target engagement, central-enzyme inhibition (e.g., ACC/FASN) can trigger compensatory pathways and, in some settings, hypertriglyceridemia; nuclear-receptor agonism shows isoform- and tissue-specific actions with class-dependent safety trade-offs; and species differences complicate extrapolation from preclinical models. These constraints support integrated pharmacodynamic and lipidomic readouts, stratification by baseline lipid signatures, and validation in diverse, aging populations.

### 7.1. Drug Targets in Lipid Intake

Many studies have shown that lipid metabolism plays a critical role in the development of various neurodegenerative disorders, particularly Alzheimer’s disease (AD) and Parkinson’s disease (PD). However, the exact mechanisms by which abnormal lipid metabolism leads to these conditions remain unclear. Targeting proteins such as fatty acid synthase (FASN), diacylglycerol O-acyltransferase 1 (DGAT), and ATP-citrate lyase (ACLY) has shown promise in alleviating some symptoms of neurodegenerative diseases. Further research is urgently needed to understand these mechanisms better and develop effective treatments [120]. First-in-class FASN inhibitors (e.g., TVB-2640) and thyroid hormone receptor-β agonists have reduced hepatic fat and improved histology in early-phase studies, whereas pan-PPAR agonists such as lanifibranor report histologic benefits with class-specific safety considerations. These agents illustrate both the promise and constraints of targeting central lipid enzymes or nuclear receptors and support biomarker-guided selection of patients with dominant lipogenic or inflammatory signatures.

A recent study indicated that CD36-mediated free FA uptake is crucial for hematopoietic stem cells (HSCs) during acute infections. This uptake triggers HSCs to shift their metabolism from anaerobic glycolysis to fatty acid β-oxidation, thereby meeting the increased energy demands necessary for HSC expansion and differentiation [121]. Inhibiting MAGL has been found to decrease inflammation and neurodegeneration significantly [122]. Furthermore, MAGL is often overexpressed in several cancers, mainly breast cancer, and is closely linked to cancer cell proliferation [123]. As a result, developing small-molecule inhibitors that target MAGL could offer promising therapeutic opportunities for treating both neurological disorders and cancer. Cholesterol is absorbed in the small intestine through the Niemann–Pick C1-like 1 (NPC1L1) receptor and taken up by cells using low-density lipoprotein receptors (LDLR) for LDL-cholesterol (LDL-C) [124]. NPC1L1 is the key transporter for intestinal cholesterol uptake and a validated target of ezetimibe, which reduces NPC1L1-mediated sterol absorption and lowers LDL-C by approximately 18–25% when added to statins, with proven cardiovascular benefit and good tolerability in large outcome trials [125,126]. Three leading types of cholesterol-lowering drugs are available: statins, which reduce the body’s cholesterol production; NPC1L1 inhibitors, which prevent cholesterol absorption in the intestine; and PCSK9 inhibitors, which aid in recycling LDL receptors more efficiently. Each of these drug classes targets a different part of cholesterol metabolism to lower cholesterol levels effectively [124]. Ezetimibe inhibits intestinal and hepatic cholesterol absorption by selectively blocking NPC1L1 internalization [125]. In post-ACS patients, adding ezetimibe to statin therapy modestly reduced major cardiovascular events versus statin alone [127].

#### 7.1.1. Drug Targets in Lipid Synthesis

SLC25A1 is a transporter protein that moves citrate from the mitochondrial matrix to the cytosol. This protein is associated with various diseases, including myasthenic syndrome [128]. CTPI-2 is a new, third-generation inhibitor of SLC25A1, followed by the first-generation benzene-tricarboxylate and the second-generation CTPI-1. In vitro studies have demonstrated that CTPI-2 can significantly reduce obesity caused by a high-fat diet and show promising antitumor effects. These findings suggest that CTPI-2 has potential as a novel SLC25A1 inhibitor with promising prospects for advancing to clinical research [129].

Overexpression of ACLY is linked to various metabolic diseases, including atherosclerosis, hyperlipidemia, and cancer. Due to its crucial physiological roles, there has been growing interest in developing small-molecule inhibitors targeting ACLY, especially following the determination of its tetrameric structure [130]. The second class of ACLY inhibitors primarily targets the citrate binding site. Notable examples include Hydroxycitric acid, SB-204990, NDI-091143, and MEDICA 16. Hydroxycitric acid, the first ACLY inhibitor discovered from a natural source, works by competitively binding to the citrate binding site, thereby inhibiting ACLY activity [131].

FASN is crucial for synthesizing fatty acids from acetyl-CoA and malonyl-CoA. Several FASN inhibitors targeting the β-ketoreductase domain have been advanced, with some progressing to clinical trials. Notable examples include BI-99179 from Boehringer Ingelheim and TVB-2640 (also known as Denifanstat) from Sagimet Biosciences. Among these, TVB-2640 is the most extensively researched. Studies have shown that TVB-2640 can reduce de novo fatty acid synthesis by up to 90% in adults with obesity and insulin resistance [132]. Because ACC inhibition can raise plasma triglycerides despite hepatic fat reduction, monitoring is advised and mitigation with fibrates may be considered in selected patients under specialist care [133,134]. Given pathway inter-dependencies, combination strategies that pair LDL-centric therapy with agents modulating de novo lipogenesis or sphingolipid signaling may be most effective in phenotypes enriched for ceramides, DAGs, or hepatic lipogenesis, particularly when guided by baseline lipidomic profiles.

#### 7.1.2. Drug Targets in Lipid Oxidation

Free FAs are activated by acyl-CoA synthetase (ACSL) to form acyl-CoA, which is essential for energy production through the oxidation of long-chain fatty acids in cardiac contraction. Impairment of ACSL1 can result in the buildup of harmful lipids that jeopardize heart function. Restoring ACSL1 activity through overexpression could potentially normalize the activation and oxidation of long-chain fatty acids, making it a promising strategy for treating heart failure [135]. For decades, CPT1 has been a key target in drug development. Researchers have aimed to create activators of CPT1 to enhance the entry of fatty acids into mitochondria, promoting oxidative metabolism and reducing fat accumulation. Conversely, small-molecule inhibitors of CPT1 have potential applications in treating other diseases. Since fatty acid oxidation (FAO) requires more oxygen than sugar metabolism, inhibiting FAO can lower cellular oxygen demands and provide protection in certain conditions. Blocking CPT1 has also shown promise in inhibiting the growth of various tumors [136].

FAO takes place in both mitochondria and peroxisomes. Medium and long-chain fatty acids are mainly broken down in the mitochondria [137], while very long-chain fatty acids (VLCFAs, those with 22 or more carbon atoms) are partially metabolized in peroxisomes. The ATP-binding cassette sub-family D member 1 (ABCD1) is a key transporter on the peroxisomal membrane that moves VLCFAs from the cytoplasm into the peroxisome. When ABCD1 malfunctions, VLCFAs build up in the cytoplasm, causing metabolic stress and leading to a condition called X-linked adrenoleukodystrophy (X-ALD). Recent research has clarified the structure of ABCD1 and its role in substrate recognition and transport, offering a deeper understanding of X-ALD [138]. This rare neurodegenerative disease mainly impacts young children, leading to a gradual and irreversible damage to neurological function, often resulting in early death.

**Table 2:** Potential pharmacological targets and inhibitors targeting lipid metabolism.

Target	Function	Inhibitors/ Modulators	Therapeutic Applications
HMG-CoA Reductase [139]	Rate-limiting enzyme in cholesterol synthesis	Statins (e.g., Atorvastatin, Simvastatin)	Hypercholesterolemia, Cardiovascular Disease
PCSK9 [140]	Regulates LDL receptor degradation	PCSK9 Inhibitors (e.g., Alirocumab, Evolocumab)	Hypercholesterolemia, Atherosclerosis
ATP Citrate Lyase (ACL) [141]	Catalyzes the conversion of citrate to acetyl-CoA	Bempedoic Acid	Hypercholesterolemia
Acetyl-CoA Carboxylase (ACC) [142]	Catalyzes the first step in fatty acid synthesis	ACC Inhibitors (e.g., Firsocostat)	Non-Alcoholic Fatty Liver Disease (NAFLD), Obesity
Farnesoid X Receptor (FXR) [143]	Regulates bile acid, lipid, and glucose metabolism	FXR Agonists (e.g., Obeticholic Acid)	NAFLD, Primary Biliary Cholangitis (PBC)
Diacylglycerol Acyltransferase (DGAT) [144]	Catalyzes the final step in triglyceride synthesis	DGAT Inhibitors (e.g., Pradigastat)	Obesity, Hypertriglyceridemia
Microsomal Triglyceride Transfer Protein (MTTP) [145]	Assists in the assembly and secretion of lipoproteins	Lomitapide	Homozygous Familial Hypercholesterolemia
NPC1L1 [125,127]	Intestinal cholesterol uptake via endocytosis	Ezetimibe	Hypercholesterolemia; adjunct to statin; outcome benefit post-ACS

Despite promising target engagement, several lipid-directed agents illustrate important translational constraints. Inhibition of central enzymes such as ACC or FASN can trigger compensatory pathways and, in some settings, hypertriglyceridemia or steatosis, which necessitates careful monitoring and combination strategies. PPAR agonists show isoform- and tissue-specific actions with heterogeneous efficacy and adverse effect profiles, and species differences in bile acid and lipid handling complicate extrapolation from rodent models. These examples emphasize the need for integrated pharmacodynamic, lipidomic, and safety readouts, stratification by baseline lipid signatures, and validation in diverse, aging human populations before broad implementation.

### 7.2. Translational and Clinical Considerations

Translating lipid–centric mechanisms into practice requires aligning patient heterogeneity with drug–class strengths and limitations. This has been illustrated by recent regulatory and trial experiences in MASH/NASH and obesity–related metabolic disease [146,147]. The FDA’s accelerated approval of the THR–β agonist resmetirom for noncirrhotic MASH with moderate–to–advanced fibrosis (F2–F3) was based on histology–based surrogate endpoints, with post–marketing requirements to confirm clinical benefit, which constrains endpoint selection, biopsy versus noninvasive test strategies, and payer adoption across diverse care settings [148].

Inter-individual variability in weight loss and metabolic improvements with GLP–1–based therapies is substantial and withdrawal studies show that benefits regress toward baseline with treatment cessation. This highlights the chronic nature of obesity care and also the importance of adherence, maintenance plans and combination strategies with lifestyle interventions [146,147]. Mechanism–specific safety trade–offs can complicate bedside use. For example, hepatic ACC inhibition reduces liver fat and NASH activity but frequently elevates circulating triglycerides, necessitating monitoring and, when suitable, mitigation with fibrates in combination regimens [133,134]. For nuclear receptor modulation, pan–PPAR activation has shown histologic improvements in MASH. Yet historical isoform–selective agents exhibited mixed efficacy and class–specific adverse effects, underscoring the need for individualized benefit–risk assessment and careful patient selection [32,51].

Real–world populations bring complexity beyond trial cohorts, including aging–related sarcopenic risk, cardiometabolic multimorbidity and polypharmacy. All of these can alter pharmacodynamics, safety margins as well as adherence trajectories for both pharmacologic and lifestyle interventions [149]. To improve generalizability and equity, future trials should stratify by sex, age, adiposity patterning and comorbidity clusters. And this should be done while integrating lipidomic and multi-omic biomarkers to match pathway lesions (e.g., de novo lipogenesis versus sphingolipid accrual) with therapeutic axes and to prospectively identify responders and non-responders [52,149].

To support clinical decision-making, we propose a pragmatic pathway-to-phenotype framework that aligns dominant lipid abnormalities with intervention strategies. Atherogenic dyslipidemia with elevated LDL-C and non-HDL-C is best targeted by high-intensity statins, with the addition of ezetimibe or PCSK9 inhibitors in patients who have residual risk or intolerance [140]. PCSK9 inhibition on top of statins further lowered LDL-C and reduced events [150]. MASLD/MASH driven by increased de novo lipogenesis and hepatic triglyceride accumulation may benefit from ACC and FASN inhibition, THRβ agonists, GLP-1/GIP receptor agonists, and structured weight loss interventions [133,134]. Ceramide- and DAG-enriched phenotypes associated with insulin resistance support therapeutic focus on adipose tissue health, sphingolipid modulation where feasible, and incretin-based therapies. In older adults with sarcopenic obesity, weight loss approaches must be combined with resistance exercise and adequate protein intake to preserve lean mass and physical function. This pathway-to-phenotype approach emphasizes matching interventions to lipid species, tissue context, and age-related vulnerability, rather than relying on single metrics such as LDL-C alone. Outcome trials including IMPROVE-IT (ezetimibe + statin) and FOURIER/ODYSSEY (PCSK9 inhibitors + statin) have shown that adding non-statin LDL-C-lowering therapies to statins further reduces major atherosclerotic events with acceptable safety profiles [127,150,151,152,153].

### 7.3. Lifestyle Interventions: Diet and Exercise as Modulators of Lipid Metabolism and Signaling

Lifestyle interventions modulate several nodes in Figure 2 simultaneously. Diets emphasizing unsaturated fats and fiber while limiting industrial trans fats and excess fructose lower hepatic substrate influx and DNL; a sustained 5–10% weight reduction in individuals with obesity or MASLD/MASH improves hepatic steatosis, triglycerides, and insulin sensitivity; and 150–300 min/week of moderate-intensity or 75–150 min/week of vigorous-intensity aerobic activity, plus 2–3 resistance-training sessions weekly, improves triglyceride-rich lipoproteins, HDL function, and skeletal-muscle FAO. In older adults or those with sarcopenic obesity, caloric restriction should be paired with adequate protein intake and resistance training to preserve lean mass and function. Managing lipid and lipoprotein disorders, as well as obesity, heavily relies on lifestyle changes. These changes are necessary for both primary and secondary prevention. For people with high cholesterol, recommended lifestyle alterations include adopting a diet low in saturated and trans fats, eating functional foods rich in bioactive compounds like fiber, antioxidants, and plant sterols, engaging in regular exercise, and maintaining a healthy weight. Current dietary regulations emphasize reducing saturated and trans fats and replacing them with mono- and polyunsaturated fats. Personalized approaches that consider individual food preferences and long-term dietary strategies are necessary to optimize lipid profiles. However, given the complexity of personal lifestyle choices, there’s considerable variability in outcomes across studies. Strong evidence associates high trans fatty acid intake with an increased risk of coronary heart disease (CHD), mainly due to their harmful impact on blood lipid profiles [154].

A meta-analysis of 60 controlled trials conducted by Mensink et al. found that replacing carbohydrates with various types of saturated fatty acids (SFAs) led to increases in both LDL-C and HDL-C levels, with the exception of stearic acid [155]. Among the SFAs studied, myristic and palmitic acids raised LDL-C and HDL-C levels similarly without significantly changing the TC/HDL-C ratio. In contrast, lauric acid had the most pronounced effect on increasing LDL-C and HDL-C levels, which resulted in a reduced TC/HDL-C ratio. Another study involving overweight men examined how different levels of carbohydrate and saturated fat intake, along with weight loss, influenced lipid profiles. During the weight-maintenance phase, participants on low-carbohydrate diets saw notable reductions in triglycerides (TG) and the concentrations of small and very small LDL particles. In contrast, those following higher-carbohydrate diets experienced only minor changes in these lipid markers [156]. These findings suggest that the content of dietary fat might not significantly affect atherogenic dyslipidemia. Instead, carbohydrate metabolism seems to influence the quality of LDL particles rather than their quantity, with low-carbohydrate diets potentially improving LDL quality. However, lower-fat and higher-carbohydrate diets might lead to greater reductions in LDL-C levels, especially in individuals with pattern B. The impact of high versus low carbohydrate diets on cardiovascular health is still not fully understood. Tailoring dietary strategies to individual needs, preferences, and metabolic or genetic factors is likely to yield the most lasting and beneficial effects on overall lipid profiles. A 2017 pooled analysis of over 37,000 adults across nine population-based studies examined the relationships between leisure-time physical activity (PA), low HDL-C levels, and mortality. The study found that individuals who followed PA guidelines (at least 150 min per week of moderate-intensity activity, 75 min per week of vigorous-intensity activity, or a combination) and had normal HDL-C levels experienced no increased risk of all-cause mortality. Interestingly, those who also followed PA guidelines but had low HDL-C levels did not face an elevated mortality risk either. However, individuals who did not meet PA guidelines and had either normal or low HDL-C levels had a higher risk of all-cause mortality. The hazard ratios for cardiovascular disease mortality were similar, though with wider confidence intervals. These results suggest that maintaining leisure-time physical activity is beneficial for those with low HDL-C, even if they do not experience the HDL-raising effects of exercise [157].

A community intervention trial involving Spanish adults with a mean age of 65 years (77% women) evaluated the impact of a nine-month supervised physical activity (PA) program. The intervention included 120 min per week of walking (396 METs/min/week) and monthly sociocultural gatherings. By the end of the study, participants in the intervention group saw significant reductions in total cholesterol (−10.1 mg/dL), LDL cholesterol (−9.1 mg/dL), and systolic blood pressure (−6.6 mmHg) compared to the control group. Two years after the program ended, the intervention group had a notably lower incidence of adverse cardiovascular events (3% vs. 11%) and higher adherence to regular PA (73% vs. 27%) compared to the control group [158]. A 2014 review by Mann et al. examined the effects of aerobic exercise training (AET), resistance training (RT), or a combination of both on cholesterol levels, analyzing data from 13 original studies and two review articles. The review confirmed that regular physical activity positively impacts cholesterol levels and provided evidence-based recommendations for exercise prescriptions. For individuals with dyslipidemia, the review suggests increasing physical activity to more than 30 min per day, five days a week. It recommends engaging in prolonged moderate-intensity AET at 70–80% of heart rate reserve, progressing to 85% of maximum heart rate, combined with moderate-to-high-intensity RT at 75–85% of one-repetition maximum [159]. Exercise can lead to alterations in the lipid composition of cell membranes, influencing their fluidity and overall cellular function. Additionally, exercise can alter the cellular and circulating lipid environment, which affects various signaling pathways in the body.

## 8. Discussion

Alterations in lipid metabolism are also linked to critical features of aging, including epigenetics. These findings indicate that lipid metabolism is a major player in biological processes, contributing significantly to aging and age-related diseases. Although current evidence suggests a connection between lipid metabolism and longevity, more research is needed to address remaining questions. In the future, studies should focus on understanding how lipid-related interventions can extend lifespan, particularly using vertebrate models to validate findings from non-vertebrate organisms like C. elegans. This review offers a comprehensive overview of the complex roles of lipid metabolism and signaling in health, aging, and metabolic diseases. We explored how closely regulated lipid pathways ensure energy homeostasis and signal cascades, while disruptions contribute to a spectrum of disorders comprising obesity, cardiovascular disease, and neurodegeneration. The convergence of lipid metabolic and signaling pathways in response to stress, aging, and disease strengthens their integrative biological impact. Essentially, this review highlights the age-related decline in lipid metabolic efficiency, described by modulated lipogenesis, impaired lipid clearance, and changes in lipid storage and signaling dynamics. These age-related alterations are associated with increased susceptibility to chronic metabolic disorders, indicating lipid dysfunction as both a driver and a biomarker of aging.

From a therapeutic perspective, recent advances feature the potential of targeting specific enzymes and receptors implicated in lipid pathways. Pharmacological interventions, such as FASN, ACC, and MAGL inhibitors, are being actively studied, while lifestyle strategies—mainly customized diets and physical activity—offer scalable and non-invasive options to mitigate lipid-associated risks. Nonetheless, the translation of these findings into clinical practice remains challenging due to the complexity of lipid signaling, systemic variability, and context-dependent outcomes. Future research should focus on integrating lipidomics with genomics and transcriptomics to unravel individualized lipid profiles and therapeutic susceptibilities. Multidisciplinary interventions, including systems biology, precision nutrition, and molecular pharmacology, are crucial for developing targeted approaches that address the root causes of lipid-driven diseases.

## 9. Conclusions

Lipid metabolism and signaling are fundamental to the regulation of cellular homeostasis, energy balance, and systemic physiological functions. This review has demonstrated the multifunctional roles of lipids, not only as structural and energetic components but also as dynamic signaling molecules that network with major pathways such as insulin signaling, mTOR, PPARs, and AMPK. These interactions are specifically significant in the framework of metabolic disorders where aberrant lipid accumulation, chronic inflammation, and altered lipid signaling contribute to disease pathogenesis.

The interaction between lipid metabolism and lipid-mediated signaling becomes increasingly complex with aging. Age-related impairments in lipid turnover, mitochondrial function, and signal transduction exacerbate receptiveness to insulin resistance, cardiovascular disease, and neurodegenerative conditions. Furthermore, changes in lipid composition and distribution during aging damage membrane integrity and receptor function, amplifying metabolic disruption. Therapeutically, targeting enzymes like ACC, FASN, and lipid-sensitive receptors such as PPARs or GPCRs provides promising avenues for metabolic intervention. Advances in lipidomics, transcriptomics, and pharmacogenomics are paving the way for precision medicine options that can stratify patient populations based on lipid signatures and individual responses to treatment. Lifestyle modifications, including caloric restriction, tailored diets, and exercise, remain necessary adjunct strategies that can synergistically improve lipid profiles and signaling outcomes. Therefore, a systemic knowledge of lipid metabolism and signaling is important for deciphering the pathophysiology of metabolic diseases and age-associated impairments. Continued research into the regulatory interactions and molecular targets within these lipid pathways will not only deepen our biological understanding but also unlock novel therapeutic strategies to deal with the growing global burden of metabolic disorders. Key unresolved questions include how to causally link specific lipid species and spatially resolved lipid niches to defined clinical phenotypes, how to safely manipulate central lipid enzymes in multimorbid and older patients, and how to embed lipidomic profiling into routine care. Addressing these gaps will enable mechanism-based prevention and treatment strategies that align lipid signatures, organ involvement, and therapeutic intensity, and will improve risk stratification in obesity- and age-associated metabolic disease.

## Figures and Tables

**Table 1:** Classification of lipid metabolism disorders.

Category	Disorder Name	Primary Lipid Involved	Key Enzyme/Protein Affected	Clinical Manifestations
Hyperlipidemias [64]	Familial Hypercholesterolemia	Cholesterol	LDL Receptor	Premature atherosclerosis, xanthomas
	Familial Combined Hyperlipidemia	Cholesterol, Triglycerides	Multiple genes involved	Elevated LDL and triglycerides, risk of CHD
	Hypertriglyceridemia	Triglycerides	LPL or ApoC-II deficiency	Pancreatitis, xanthomas, hepatosplenomegaly
Hypolipidemias [65]	Abetalipoproteinemia	Cholesterol, Triglycerides	Microsomal triglyceride transfer protein (MTTP)	Fat malabsorption, retinal degeneration, neuropathy
	Hypoalphalipoproteinemia	HDL	ApoA-I deficiency	Low HDL levels, increased risk of atherosclerosis
Lysosomal Storage Disorders [66]	Gaucher Disease	Glucosylceramide	Glucocerebrosidase	Hepatosplenomegaly, bone crises, neurological symptoms
	Niemann-Pick Disease	Sphingomyelin, Cholesterol	Sphingomyelinase (Types A, B)	Hepatosplenomegaly, neurodegeneration
Peroxisomal Disorders [67]	Zellweger Syndrome	Very-long-chain fatty acids	Peroxisome biogenesis	Craniofacial dysmorphism, liver dysfunction
Fatty Acid Oxidation Disorders [68]	Medium-Chain Acyl-CoA Dehydrogenase Deficiency (MCADD)	Medium-chain fatty acids	Medium-chain acyl-CoA dehydrogenase	Hypoglycemia, lethargy, liver dysfunction
Cholesterol Metabolism Disorders [69]	Smith-Lemli-Opitz Syndrome	Cholesterol	7-Dehydrocholesterol reductase	Developmental delay, dysmorphic features
	Sitosterolemia	Plant sterols	ABCG5/ABCG8	Tendon xanthomas, premature atherosclerosis

## Data Availability

No new data were created or analyzed in this study.

## Abbreviations

The following abbreviations are used in this manuscript:

ACC	Acetyl-CoA carboxylase
ACSS2	Acyl–CoA synthetase short–chain family member 2
ACLY	ATP-citrate lyase
ACS/ACSL	Acyl-CoA synthetase/Acyl-CoA synthetase long-chain
AMPK	AMP-activated protein kinase
AET	Aerobic exercise training
ALA	α-Linolenic acid
ApoA-I	Apolipoprotein A-I
ApoC-II	Apolipoprotein C-II
ATGL	Adipose triglyceride lipase
CIC/SLC25A1	Mitochondrial citrate carrier
CPT-1	Carnitine palmitoyltransferase 1
cAMP	Cyclic adenosine monophosphate
CREB	cAMP response element-binding protein
DAG	Diacylglycerol
DNL	De novo lipogenesis
DGAT	Diacylglycerol acyltransferase
FA	Fatty acid
FAO	Fatty acid oxidation
FASN/FAS	Fatty acid synthase
FFA	Free fatty acid
FXR	Farnesoid X receptor
GPL	Glycerophospholipid
HDL	High-density lipoprotein
HFD	High-fat diet
HSC	Hematopoietic stem cell
IGF-1	Insulin-like growth factor 1
IIS	Insulin/IGF-1 signaling
IP3	Inositol trisphosphate
IRS	Insulin receptor substrate
JNK	c-Jun N-terminal kinase
KGDHC	(Not used—ignore)
Krebs cycle/TCA	Tricarboxylic acid cycle
LD/LDs	Lipid droplet(s)
LDL	Low-density lipoprotein
LPL	Lipoprotein lipase
ME	Malic enzyme
MET	Metabolic equivalent of task
mTOR/mTORC1/mTORC2	Mechanistic target of rapamycin (complex 1/2)
MUFAs	Monounsaturated fatty acids
NAEs	N-acylethanolamides (e.g., anandamide)
NAFLD	Non-alcoholic fatty liver disease
NASH	Non-alcoholic steatohepatitis
NPC1L1	Niemann–Pick C1-like 1
OXPHOS	Oxidative phosphorylation
PA/PPL	Physical activity/Postprandial lipemia
PCSK9	Proprotein convertase subtilisin/kexin type 9
PDK1	Pyruvate dehydrogenase kinase 1
PFA	Paraformaldehyde (in immunohistochemistry, if used)
PIP_2_/PIP_3_	Phosphatidylinositol-4,5-bisphosphate/Phosphatidylinositol-3,4,5-trisphosphate
PLIN1/2/5	Perilipins
PPAR (α, γ-1, γ-2, δ)	Peroxisome proliferator-activated receptor
SCAP	SREBP cleavage-activating protein
SCD	Stearoyl-CoA desaturase
SCFA	Short-chain fatty acid
SFA	Saturated fatty acid
SREBPs	Sterol regulatory element-binding proteins
TG	Triglyceride
TVB-2640	Denifanstat (Fatty acid synthase inhibitor)
TCA	Tricarboxylic acid cycle
VLCFA	Very-long-chain fatty acid
VLDL	Very low-density lipoprotein
WAT	White adipose tissue
